# Top 100 most-cited articles on hemorrhoids: A bibliometric analysis and visualized study

**DOI:** 10.3389/fsurg.2022.1021534

**Published:** 2022-11-11

**Authors:** Zhaochu Wang, Xuxiong Wu, Yang Li, Juan Huang, Rong Shi, Jing Wang

**Affiliations:** Department of Anorectal Surgery, The Affiliated People's Hospital of Fujian University of Traditional Chinese Medicine, Fuzhou, China

**Keywords:** bibliometric, visualized study, hemorrhoids, Milligan-morgan hemorrhoidectomy, stapled hemorrhoidectomy

## Abstract

**Background:**

Hemorrhoids have a significant incidence in people and are becoming a common public health problem. This study provides a bibliometric and visualized analysis of the most influential literature in the field. The aim is to reveal trends in the field of hemorrhoids and to provide a reference for researchers.

**Methods:**

The 100 most frequently cited studies in the field of hemorrhoids were collected from the Web of Science(WOS), and were analyzed in terms of the annual publication, types of literature, countries, institutions, authors, journals, and keywords. During the study, we used a combination of VosViewer, Carrot2, Microsoft Excel, and Tableau tools to better present the visual information.

**Results:**

A total of 4,481 articles were retrieved, of which 3,592 were of the Article and Review types, among which we selected the 100 most frequently cited. A large amount of highly cited literature on hemorrhoid surgery emerged from 1990 to 2010, and the interest of researchers in hemorrhoid surgery seems to have waned after 2010. The sources of highly cited literature in the field of hemorrhoids are predominantly Western, with the United States. and the United Kingdom accounting for almost half of the publications worldwide. However, countries with higher prevalence populations do not have significant research on hemorrhoids. St. Mark's Hospital has published the largest number of influential articles in the field of hemorrhoid disease. Kamm MA and Phillips RKS are the most authoritative authors in the field. *Diseases of the Colon & Rectum* and the *British Journal of Surgery* are the most influential journals in this field. The highly cited literature covers a wide range of disciplines, with Thomson's classic “The nature of hemorrhoids” receiving the most attention among the studies focusing on hemorrhoids. Keyword and clustering analysis revealed that The most famous focus in the field of hemorrhoid research is the evolution of stapled hemorrhoidectomy (SH) and Milligan-morgan hemorrhoidectomy (MMH).

**Conclusions:**

This study is the first to explore developments in the field of hemorrhoids, and it helps surgeons quickly understand global trends in the field of hemorrhoids. In recent years, the development of hemorrhoids seems to have hit a bottleneck, with scholarly interest in the field of waning, especially in surgery Procedures. The theory of inferior anal cushion migration has proven to be the most influential theory in the field, but after studies based on SH and MMH, more high-quality evidence is needed to continue advancing the field of hemorrhoids. The results of this study are intended to add to the attention and interest of scholars in this area and provide a reference for further research.

## Introduction

Research shows that more than one-third of people have hemorrhoids that are found during colonoscopy (1–3); however, flexible endoscopy cannot diagnose asymptomatic hemorrhoids (4). This means that hemorrhoids have more potential to develop in the population and are one of the most common diseases in the world.

While the main symptoms of internal hemorrhoids are painless bleeding and intermittent prolapse, thrombosed external hemorrhoids can cause severe pain to the patient (5). Thomson's research in 1975 established the basis for the theory of inferior displacement of the anal cushion, which suggests that hemorrhoids develop due to congestion, hypertrophy, and prolapse of the anal cushion (6). A 2018 guideline suggests that improving diet and lifestyle habits is the first-line therapy for hemorrhoids, while there are many medication options (7). Conservative treatment usually fails, and patients with stage III and IV hemorrhoids can consider surgery. Surgery, outpatient treatment, and medication are effective treatments for hemorrhoids, but clinically they have more classifications and options (7, 8).

Despite the enormous economic burden and distress hemorrhoids cause society, they still receive little attention (9). The lack of understanding of hemorrhoids by many physicians, coupled with the fact that hemorrhoids often have overlapping signs and symptoms with other anorectal conditions, means that hemorrhoids are often incorrectly evaluated (10). In the United States., billions of dollars are spent on hemorrhoid treatment each year, but physician misdiagnosis raises this value (5). Due to a lack of awareness, many cases of overtreatment may occur, or people may severely underestimate the dangers of symptomatic hemorrhoids (9).

Therefore, we explored the field of hemorrhoids for the first time using a bibliometric approach to identify global trends and the knowledge architecture of hemorrhoids. We hope to provide a comprehensive basis for surgeons' research and clinical work and insights into explorations in the field.

## Methods

### Bibliometric analysis and visualized study

Bibliometric analysis provides a quantitative approach to exploring a specific field to discover the dynamics and progress of a discipline (11–13). A visualized study facilitates the interpretation of data and uncovers the internal connections between them (14). During this study, we used Excel (Version 2021), VOSviewer (Version 1.6.18), Carrot2 (https://search.carrot2.org/#/workbench), and Tableau (Version 2022.2) for the bibliometric analysis and visualization of the literature. In scientific research, the act of citation is meant to endorse or critique the cited literature's results; thus, highly cited literature represents the hot spots of a field of research and the trend carriers of the discipline (15). Therefore, we selected 100 articles of great importance, including the main annual publication trends, type of literature, country, author, institution, publication, co-occurrence of keywords, and cluster analysis.

### Search strategy and literature screening

**Figure 1:**
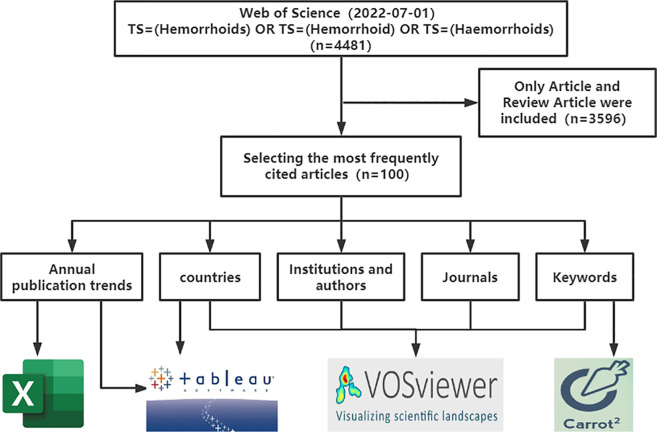
Research flowchart.

WOS is considered the most worthwhile database for bibliometric analysis because of its high quality (16, 17). We collected literature from the WOS Core Collection from 1900 to 2022, and searched for the date 2022–07–01. In developing the search strategy, we referred to the MeSH subject headings list and some older literature and finally settled on TS = (Hemorrhoids) OR TS = (Hemorrhoid) OR TS = (Haemorrhoids). After the search, we sorted all entries by citation frequency, and only original research articles and reviews were taken into account. Meeting abstract, letter, proceeding paper, and other types of materials will not be considered. Finally, we found the 100 most frequently cited articles. It is important to note that this literature contains several different fields, such as gastroenterology, surgery, and even botany, as we used a subject search, and some of the literature only mentioned hemorrhoids in the abstract and were therefore included in the study. We believe that these articles equally advance the field of hemorrhoids. The collection process of the literature was carried out independently by the two authors, and after that, after which a final agreement was reached through communication. We document the study flow in detail in Figure 1 to provide the reader with a better understanding of our work and to ensure the reproducibility of the study.

## Results

### Annual publication trends

**Figure 2:**
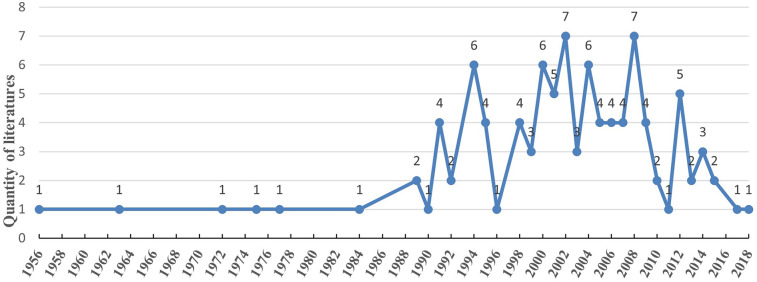
Annual publication of the top 100 most-cited articles.

**Figure 3:**
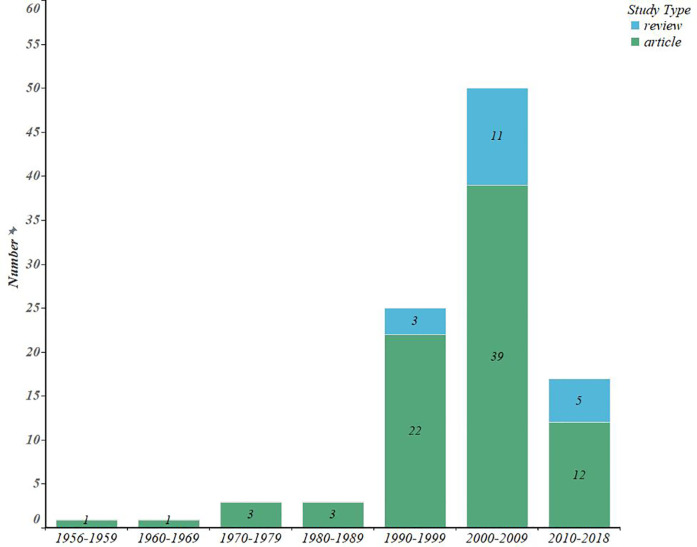
Study type histogram of the top 100 most-cited articles.

Through the search, we found 4,481 publications and saved the 100 most cited Article and Review literature. Figure 2 shows that, literature was published from 1956 to 2018, with a low point before 1990, after which the number of publications grew significantly faster, with peaks in 2002 and 2008, with seven publications. However, there were only 15 highly cited publications from 2010 to 2018, and only 1 of them was related to surgery for hemorrhoids. As shown in Figure 3, the number of Articles in the top 100 papers far exceeds that of Reviews, and most of the two literature types are primarily concentrated in 2000–2009. Table 1 records the ten most influential publications, and we found that Anderson's 2009 article was cited much more often than the other 9.

**Table 1:** Top 10 most-cited articles.

Rank	First author	Title	Publication year
1	Anderson	Health benefits of dietary fiber	2009
2	MacLennan	The prevalence of pelvic floor disorders and their relationship to gender, age, parity and mode of delivery	2000
3	Peery	Burden of Gastrointestinal, Liver, and Pancreatic Diseases in the United States	2015
4	Callam	Epidemiology of varicose veins	1994
5	Potosky	Five-year outcomes after prostatectomy or radiotherapy for prostate cancer: The prostate cancer outcomes study	2004
6	Thomson	The nature of hemorrhoids	1975
7	Johanson	The prevalence of hemorrhoids and chronic constipation: An epidemiologic study	1990
8	Mehigan	Stapling procedure for hemorrhoids versus Milligan-Morgan hemorrhoidectomy: randomized controlled trial	2000
9	Mishra	The effect of curcumin (turmeric) on Alzheimer's disease: An overview	2008
10	Macrae	Comparison of hemorrhoidal treatment modalities. A meta-analysis	1995

### Countries

**Figure 4:**
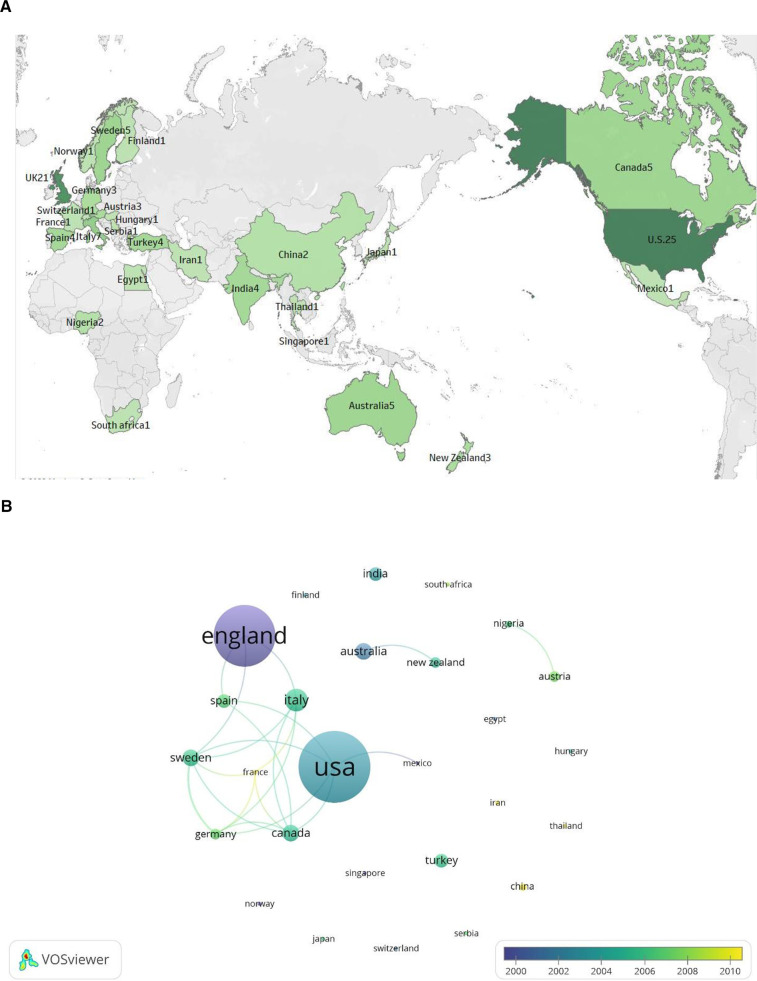
Countries of publication of the top 100 most-cited articles. (**A**) Geographical visualization of national publications, (**B**) Countries’ cooperation coexistence diagram.

We use the algorithm that comes with VOSviewer for country publication measurement to ensure the most accurate statistics are obtained. The country statistics using VOSviewer takes into account all authors, but if multiple authors in 1 article are from the same country, only one count is performed. We merged Scotland into the UK, so 30 countries were involved in publishing highly cited literature. Figure 4A shows the geographical visualization between countries, and we find that they are primarily coastal. Table 2 records the 15 countries with the highest number of publications, with the United States. (*n* = 25) and the UK (*n* = 21) having nearly the same number of highly cited articles as the other countries combined. The average number of citations for publications from Australia, New Zealand, and the United States is significant, at 275.4, 274.3, and 221.5, respectively, indicating that some of their articles are highly influential. Figure 4B shows a lack of cooperation between countries, mainly concentrated among developed Western countries.

**Table 2:** The top 15 influential countries.

Rank	Country	No.of articles	Total citations	Average Article Citations
1	United States.	25	5,537	221.48
2	United Kingdom	21	3,403	162.05
3	Italy	7	1,246	178.00
4	Australia	5	1,377	275.40
5	Canada	5	859	171.80
6	Sweden	5	775	155.00
7	India	4	471	117.75
8	Spain	4	423	105.75
9	Turkey	4	508	127.00
10	Austria	3	364	121.33
11	Germany	3	498	166.00
12	New Zealand	3	823	274.33
13	China	2	245	122.50
14	Nigeria	2	201	100.50
15	Egypt	1	177	177.00

### Institutions and authors

**Figure 5:**
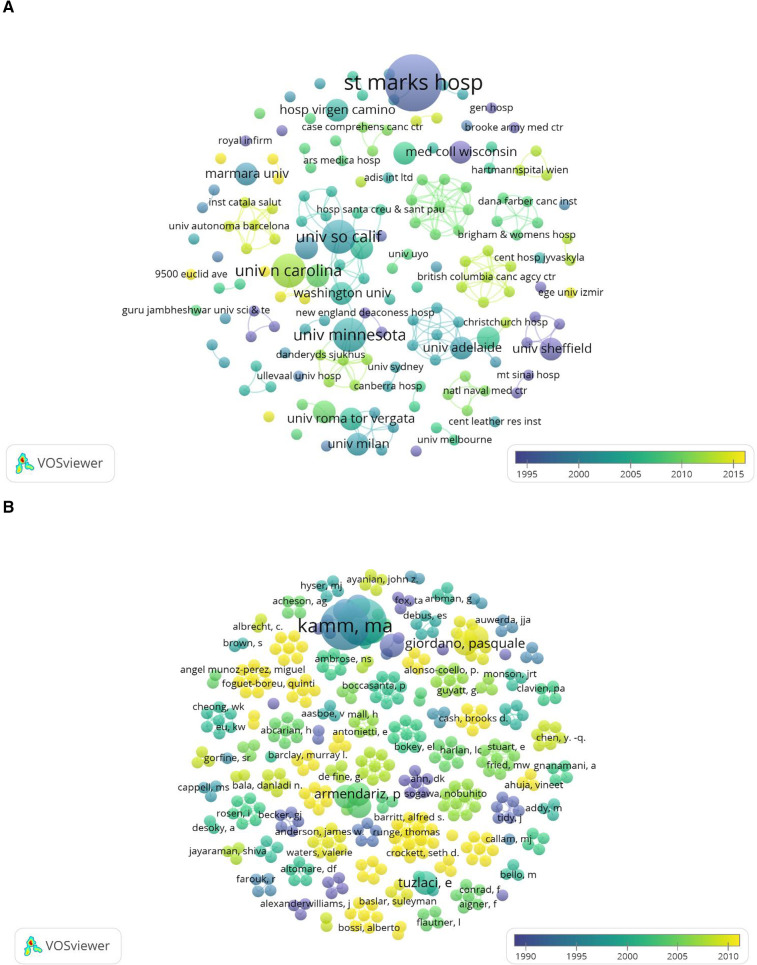
Publication status of authors and institutions of the 100 most-cited articles. **(A)** Institutions’ cooperation coexistence diagram, **(B)** authors’ cooperation coexistence diagram.

A total of 163 institutions were involved in publishing highly cited literature, and the top 10 most cited institutions are recorded in Table 3. The institution with the most publications is St. Mark's Hospital (*n* = 5) in the UK, followed by the University of Minnesota (*n* = 3), the University of North Carolina (*n* = 3), and the University of Southern California (*n* = 3), all in the United States. The partnership between the institutions can be seen in Figure 5A, which shows that St. Mark's Hospital has an early start and a significant number of high-value publications. As shown in Table 4, a total of 393 authors participated in the highly cited literature, with Kamm MA (*n* = 4), and Phillips RKS (*n* = 4) publishing the most articles, and the remaining authors all publishing only 1–2 influential papers each in the field of hemorrhoids. The collaborative relationship between the authors is shown in Figure 5B.

**Table 3:** The top 10 most-influential institutions.

Rank	Institution	No. of articles	Total citations	Average Article Citations
1	St. Mark's Hospital	5	859	171.80
2	Univ Minnesota	3	479	159.67
3	Univ North Carolina	3	818	272.67
4	Univ Southern California	3	695	231.67
5	Hospital Virgen Del Camino	2	223	111.50
6	Marmara Univ	2	258	129.00
7	Mayo Clinic	2	315	157.50
8	Med Coll Wisconsin	2	439	219.50
9	Mt Sinai Sch Med	2	228	114.00
10	Univ Adelaide	2	657	328.50

**Table 4:** The top 10 most-influential authors.

Rank	Author	No. of articles	Total citations	Average Article Citations
1	Kamm, MA	4	750	187.5
2	Phillips, RKS	4	719	179.75
3	Johanson, JF	3	531	177
4	Armendariz, p	2	223	111.5
5	Cheetham, MJ	2	347	173.5
6	Giordano, P	2	228	114
7	Gravante, G	2	228	114
8	Loder, PB	2	372	186
9	Marzo, J	2	223	111.5
10	Nicholls, RJ	2	372	186

### Journals

The 100 most cited studies **on hemorrhoids** were published in 42 journals, and Table 5 records the ten most published journals, along with their citation frequency, impact factor, and division. Among them, *Diseases of the Colon & Rectum* (*n* = 19) ranked first, followed by the *British Journal of Surgery* (*n* = 14) and the *American Journal of Gastroenterology* (*n* = 5), among others. This information will help inform the dissemination of articles in this area. Although there are few publications in *Lancet* and *Gastroenterology*, their average article citations are 220.8 and 330.3, respectively, but this does not explain the correlation between the impact factor and the number of citations.

**Table 5:** The top 10 journals containing the most cited literature.

Rank	Journal	No. of articles	Total citations	Average Article Citations	2021 IF
1	Diseases of the Colon & Rectum	19	2,724	143.4	4.412
2	British Journal of Surgery	14	2,631	187.9	11.122
3	American Journal of Gastroenterology	5	730	146.0	12.045
4	American Journal of Surgery	5	608	121.6	3.125
5	Journal of Ethnopharmacology	5	626	125.2	5.195
6	Lancet	5	1,104	220.8	202.731
7	Archives of Surgery	3	338	112.7	0
8	Fitoterapia	3	361	120.3	3.204
9	Gastroenterology	3	991	330.3	33.883
10	International Journal of Colorectal Disease	3	376	125.3	2.796

### Keywords

**Figure 6:**
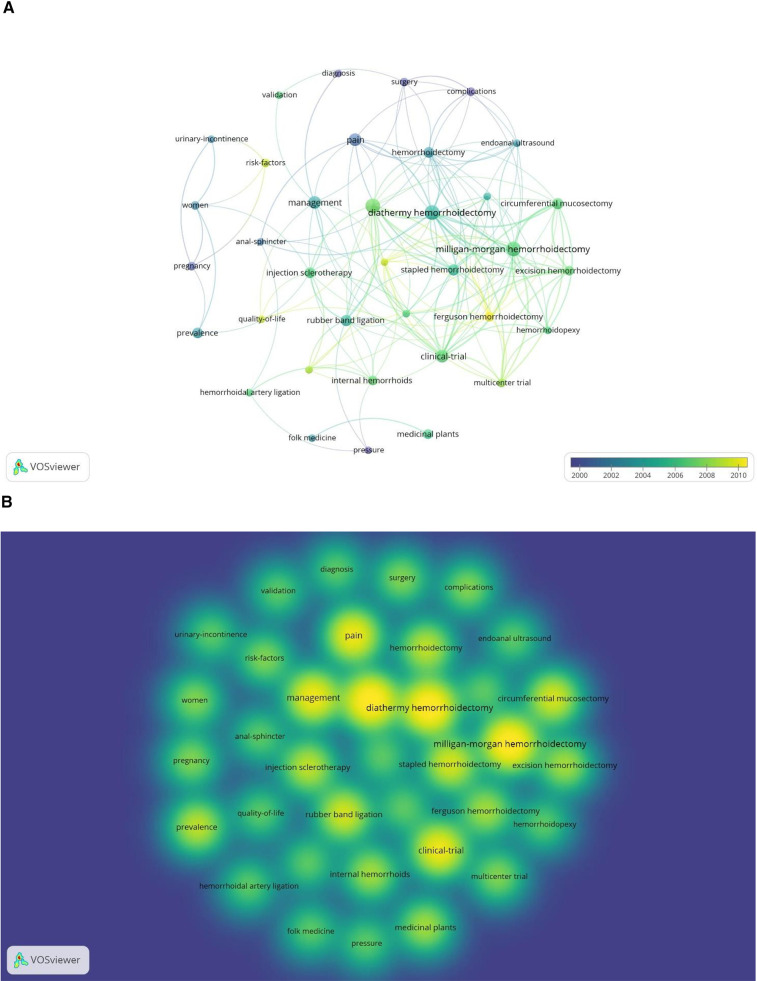
Network chart of keywords of the 100 most-cited articles. (**A**) Keyword co-occurrence chart, (**B**) Keyword heat diagram.

We performed a co-occurrence network analysis of keywords extracted from 100 documents to identify the most important keywords and their relationships. We set the keyword threshold to “3”, cleaned the data of keywords with the same meaning, and ticked to hide the keywords of “hemorrhoids,” “disease,” and “expression” with little meaning. As shown in Figure 6A, the size of each node depends on the number of keyword occurrences, the color represents the average year of keyword occurrences, and the connecting line represents two keywords appearing together in the literature.

We found that “pregnancy,” “pressure,” “surgery,” “diagnosis,” and “complications” were the early keywords, suggesting that the early focus of researchers was on the onset of hemorrhoids, diagnosis, anal pressure, and the impact of surgery on complications. “Ferguson hemorrhoidectomy,”(FH) “quality of life,” “risk factors,” and “multicenter trial” are newer keywords in the figure, suggesting that researchers may henceforth focus more on high-quality randomized controlled trials, surgical modalities, and their prognosis. Numerous keywords on hemorrhoid treatment were focused on in 2006–2008, including “conventional hemorrhoidectomy,” (CH) “diathermy hemorrhoidectomy,”(DH) “excision hemorrhoidectomy,”(EH) “SH,” “MMH,” “injection sclerotherapy,” “hemorrhoidal artery ligation,” and “medicinal plants,” thus indicating that this period was a peak in the development of hemorrhoid disease, with the addition of evidence for many treatments driving the research the boom.

**Figure 7:**
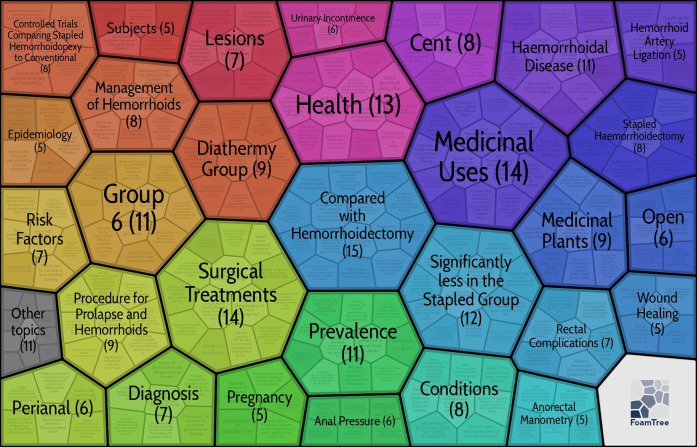
30 clustered area diagram formed by the 100 most-cited articles.

Figure 6B shows the visualization of keyword hotness, from which we can see that “MMH,” “DH”, “clinical-trial, “management,” and “pain” are the most popular keywords. These represent the most popular topics in the field of hemorrhoids. “Circumferential mucosectomy,” “rubber band ligation,” and “injection sclerotherapy” followed in popularity as the more common surgical treatments for hemorrhoids. To obtain satisfactory clustering results, we used Carrot2 to cluster the keywords of the highly cited literature on hemorrhoid disease. From Figure 7, the most important clusters are “Compared with Hemorrhoidectomy” and “Surgical Treatments,” followed by “Medicinal Uses.”

## Discussion

Shneider A defines the development of disciplines as taking place in four stages: introducing new problems, the birth of research tools, the generation of disciplinary knowledge based on tools, and the transfer of knowledge and methods (18). Even though hemorrhoids are one of the most common diseases in the world, no scholars have used bibliometric tools to uncover knowledge in the field of hemorrhoids until now. A well-established field needs extensive citation literature to support it. Although a discussion of only the highly cited literature is not representative of the field as a whole, it gives us an idea of the most influential historical contributions and the directions scholars are most concerned with (19), and reduces the redundancy generated by large samples of data.

The amount of highly cited literature was low until 1990, after which it began to increase irregularly until after 2010 when scholarly interest in the field seems to have decreased again. However, some new articles in recent years may be of significant value but have been published for a relatively short period of time and therefore have not been widely cited. This may also be one of the reasons.

The number of highly cited studies in the United States. and the United Kingdom is almost equal to that of the other countries combined, especially since most institutions with many publications are from the United States. Hemorrhoids are a disease of all humans, and the fact that most of the highly cited literature comes from Western countries may be related to economic and scientific strength, not that hemorrhoids are widespread only in Western countries. For example, Asian countries such as Israel and South Korea and African countries such as Ethiopia alomst have the highest hemorrhoid rates globally, which is unbalanced (20–22). Some cooperation exists between western countries, but academic exchanges are still lacking in most other countries. The public has become aware of the economic and work day burden that hemorrhoids place on health systems (9, 23), so we are calling on researchers to increase their attention to hemorrhoids, especially in countries whose population is that are deeply affected by hemorrhoids.

The most frequently cited article is Anderson 2009, which found that high levels of dietary fiber reduce the prevalence of hemorrhoids (24). The first ten articles also included three epidemiological reports: MacLennan 2000 concluded that hemorrhoid symptoms were positively correlated with age and fecundity (25), Peery 2015 found 4 million cases of hemorrhoids annually, accounting for the third highest number of outpatient diagnoses of gastrointestinal, liver, and pancreatic diseases in the United States (26), and Johanson 1990 found that hemorrhoids and constipation did not have a significant causal relationship (27). Unfortunately, none of the five most cited articles had hemorrhoids as a significant research target, so the most influential article in the field of hemorrhoids was Tomshen 1975 (5), which suggests that the doctrine of inferior anal cushion migration, based on this article, has the greatest influence in the current field of hemorrhoids. Also, the most popular studies were Mehigan 2000 and Macrae 1995, who compared different treatments for hemorrhoids (28, 29).

Kamm MA (*n* = 4), and Phillips RKS (*n* = 4), published the most highly cited papers in the field of hemorrhoid disease, with their studies published from 1991 to 2003. The main topics include a comparison of the efficacy of SH and DH (30, 31), the pathogenesis of hemorrhoids (32), and the effect of anal dilation on the anal sphincter (33). It was followed by Johanson JF (*n* = 3), whose studies were published from 1990 to 2006 and included: an epidemiological comparison of hemorrhoids and constipation (27), and nonsurgical treatment of hemorrhoids (34, 35). “MMH,” “DH,” and “RCT” are the most frequent keywords. MMH and DH are regarded as the same traditional hemorrhoid surgery (36), a classic procedure derived from the “Milligan” and “Morgan” of St. Mark's Hospital in the UK in 1937 (37), which was considered the gold standard for hemorrhoid surgery in the past (38). We read Citations Citing Articles to identify those keywords, and most of them are related to SH (39–41), a surgical method proposed by Longo in 1998 (42), which is also called the Procedure for Prolapse and Hemorrhoids (43), and was sometimes considered an alternative to conventional MMH (40). This can explain the second increase in the emergence of highly cited literature after 1998, implying that the impact of new surgical modalities was yielding important scientific results. We found that the comparison between SH and MMH is almost the most significant focus in the field of hemorrhoids because they are controversial, with the anastomosis procedure reducing short-term pain in patients but with problems of stool urgency and high recurrence rates in long-term follow up (44–46), so some scholars consider that MMH remains the gold standard for hemorrhoid surgery (38, 47). We found many articles on hemorrhoid surgery appearing between 1988 and 2010, but of the 15 highly cited literature after 2010, only one was related to the surgery of hemorrhoids (48). This study summarizes and updates the previous Meta-analysis for comparing 11 surgical procedures for grade III and IV hemorrhoids. The highly cited literature after 2010 includes epidemiology and guidelines for hemorrhoids and also relates to pharmacology and gastroenterology. This implies that although the comparison of SH with MMH remains the most important part of the field as a whole, the development of procedures related to hemorrhoids after 2010 may have reached a bottleneck, leading to a waning of scholarly interest in the disease. Therefore, we believe that an update of the procedures and further cross-collaboration between hemorrhoids and other fields may be the key to the revitalization of the field again. In addition, we noted some high-frequency secondary keywords: “rubber band ligation” and “injection sclerotherapy.” Bleday found that 44.8% of patients underwent rubber band ligation in 1985, and 0.7% underwent injection sclerotherapy in 1990 (49). The 2018 ASCRS guidelines strongly recommend that they are a treatment option for grades I, II, and III hemorrhoids if pharmacologic therapy fails (7), but infrared coagulation did not appear in our high-frequency keywords.

We clustered the snippet and title of the document using the Lingo algorithm based on singular value decomposition. The Lingo algorithm, proposed by Osiński S (50), differs from traditional STC clustering algorithms in that it produces many small clusters and places great emphasis on the descriptive quality of the clusters. We performed a cluster analysis of these keywords to help identify some standard labels in the literature with similar characteristics and reveal hotspots (51, 52). The two most significant clusters reiterate the focus in the field of hemorrhoids. In addition to SH and MMH, surgical modalities such as FH and Transanal hemorrhoidal dearterialization(THD) are included (48, 53). The third central cluster, Medicinal Uses, mentions many medicinal plants for hemorrhoids (54, 55), and although this literature is rarely referenced in the field of hemorrhoids, it may provide fruitful directions.

## Limitation

We have carefully considered the study's limitations and, therefore, need to describe our results more objectively. First, we designed this study to ensure the quality and integrity of the literature and to reduce the production of duplicate data by only selecting data from WOS, which, despite being the most prestigious database in the world, may still miss some studies. Second, the analysis of only the highly cited literature is not representative of the entire field; some new research has an impact, but citation frequency is a cumulative process. Third, unlike classification, clustering algorithms are a form of unsupervised deep learning, and although the computer uses the Lingo algorithm, which produces more easily decipherable clustering labels, there are still some labels whose meaning is unclear, and we usually need to focus only on the most important clusters.

## Conclusion

This study is the first bibliometric analysis of the field of hemorrhoids. Our findings suggest that despite the significant increase in highly cited literature on hemorrhoids after 1990, scholarly interest has waned significantly after 2010, especially as the highly cited literature on hemorrhoid surgery is very sparse. This does not mean that the field is no longer essential to explore, as there are some new social burdens. In addition, excellence is necessary for the development of surgery. The volume of highly cited literature in the United States. and the United Kingdom is almost equal to that of other countries combined, so more research efforts are needed to advance the field of hemorrhoid disease. Our study reveals global trends in hemorrhoid disease, with the doctrine of inferior anal cushion migration having the greatest impact on the development of hemorrhoids, and the comparison of SH and MMH is probably the most popular focus in the field, meaning that the emergence of new surgical modalities will cause dramatic changes. Researchers can quickly learn about the field through the evolution of related procedures, for example, by looking for hot spots in the latest surgical approaches, leading to more collaborations.

## Data Availability

The original contributions presented in the study are included in the article/Supplementary Material, further inquiries can be directed to the corresponding author/s.
